# Characterization of Leptin Receptor^+^ Stromal Cells in Lymph Node

**DOI:** 10.3389/fimmu.2021.730438

**Published:** 2022-01-17

**Authors:** Liwei Jiang, Mine Yilmaz, Mayuko Uehara, Cecilia B. Cavazzoni, Vivek Kasinath, Jing Zhao, Said Movahedi Naini, Xiaofei Li, Naima Banouni, Paolo Fiorina, Su Ryon Shin, Stefan G. Tullius, Jonathan S. Bromberg, Peter T. Sage, Reza Abdi

**Affiliations:** ^1^ Transplantation Research Center, Renal Division, Brigham and Women’s Hospital, Harvard Medical School, Boston, MA, United States; ^2^ Institute of Health and Medical Technology, Hefei Institutes of Physical Science, Chinese Academy of Sciences, Hefei, China; ^3^ Division of Nephrology, Boston Children’s Hospital, Harvard Medical School, Boston, MA, United States; ^4^ Biomaterials Innovation Research Center, Division of Biomedical Engineering, Department of Medicine, Brigham and Women's Hospital, Harvard Medical School, Cambridge, MA, United States; ^5^ Division of Transplant Surgery, Brigham and Women’s Hospital, Harvard Medical School, Boston, MA, United States; ^6^ Departments of Surgery and Microbiology and Immunology, University of Maryland School of Medicine, Baltimore, MD, United States

**Keywords:** lymph node, stromal cell, leptin receptor, type 2 diabetes, matrix structure

## Abstract

Lymph node (LN)-resident stromal cells play an essential role in the proper functioning of LNs. The stromal compartment of the LN undergoes significant compensatory changes to produce a milieu amenable for regulation of the immune response. We have identified a distinct population of leptin receptor-expressing (LepR^+^) stromal cells, located in the vicinity of the high endothelial venules (HEVs) and lymphatics. These LepR^+^ stromal cells expressed markers for fibroblastic reticular cells (FRCs), but they lacked markers for follicular dendritic cells (FDCs) and marginal reticular cells (MRCs). Leptin signaling deficiency led to heightened inflammatory responses within the LNs of db/db mice, leakiness of HEVs, and lymphatic fragmentation. Leptin signaling through the JAK/STAT pathway supported LN stromal cell survival and promoted the anti-inflammatory properties of these cells. Conditional knockout of the LepR^+^ stromal cells in LNs resulted in HEV and extracellular matrix (ECM) abnormalities. Treatment of ob/ob mice with an agonist leptin fusion protein restored the microarchitecture of LNs, reduced intra-LN inflammatory responses, and corrected metabolic abnormalities. Future studies are needed to study the importance of LN stomal cell dysfunction to the pathogenesis of inflammatory responses in type 2 diabetes (T2D) in humans.

## Introduction

LNs are highly specialized organs that monitor the incoming lymph continuously from organs *via* afferent lymphatic ducts. Lymph first enters the subcapsular region of a LN, then progresses through the medullary region of the LN, and leaves the LN *via* efferent lymphatic ducts (1). LNs monitor the tissue fluid exudate as a method of surveying the immune milieu of organs (2, 3). Lymphatic expansion in LNs is a cardinal manifestation of heightened inflammatory responses within the organs that they drain (4).

Key to the function of LNs is the presence of specialized stromal cells that not only provide the scaffold for the LN, but also perform a plethora of physiological functions (3, 5). The majority of stromal cells within LNs are FRCs, which are a podoplanin (PDPN)^+^CD31^–^ populations. FRCs are thought to originate from mesenchymal stem cells in adipose tissue. They build a scaffold within the LN upon which incoming T cells that have entered *via* HEVs can crawl to meet dendritic cells (DCs) (6). The homing of immune cells to the LN is promoted by the secretion of chemokines, such as CCL19, by FRCs (5). FRCs possess various immunoregulatory molecules, which can amplify or dampen immune responses (7, 8). Pericytes, which are stromal cells, are critically important to the integrity of the microvasculature (9, 10). Pericytes reside within the vicinity of the basement membranes of endothelial cells (11, 12). Both the lymphatics and HEVs of the LNs are among some of the most active vasculatures in the body. On a daily basis, millions of T cells home to the LNs *via* HEVs, requiring the presence of strong supporting cells for the integrity of HEVs. Furthermore, the lymphatic vessels in the LNs must be capable of expansion in response to any ongoing injuries within the organs (13, 14). Substantial progress has been made in deciphering more deeply the stromal population within the LNs; however, many unanswered questions remain. Despite the overwhelming body of work on the cellular and humoral effector players of inflammatory responses in T2D, these studies have not studied LNs, critical sites for immune activation and regulation, in depth.

Multiple immune processes are involved in the pathogenesis of T2D (15, 16). Pro-inflammatory cellular and humoral responses have been shown to play key roles in the pathogenesis of T2D (17). Elevated leptin levels are associated with insulin resistance and T2D development (18). Leptin exerts its biological functions through a cell-surface LepR that is a member of the type I cytokine receptor family (19). Recognition of the contribution of leptin to inflammation originated by demonstrating extensive expression of LepR by leukocytes (20–23). Leptin is known for its role regulating T cell immunity (24, 25). However, the role of Leptin in regulating the function of stromal cells and its potential implication in regulating the inflammatory milieu of LN remain to be further examined. Furthermore, lymphatic vessel dysfunction is an emerging component of metabolic diseases implicated in obesity (26, 27). However, the existence of lymphatic and HEV dysfunction in T2D has not been investigated.

In this study, we identified leptin receptor-expressing (LepR^+^) stromal cells, located predominantly in the vicinity of HEVs and lymphatic vessels inside LNs. Our data showed that leptin signaling plays a critical role in maintaining the integrity and proper functioning of HEVs and lymphatic vessels in murine LNs. Leptin signaling deficiency leads to lymphatic fragmentation, HEV leakiness, and increased inflammatory responses within LNs. Treatment with leptin fusion protein restored the anti-inflammatory properties and microarchitecture of LNs in leptin-deficient ob/ob mice. These studies shed new light on the potential importance of LNs in regulation of inflammatory responses seen in metabolic syndromes, such as T2D.

## Results

### Localization and Characterization of LepR^+^ Cells in LNs

**Figure 1:**
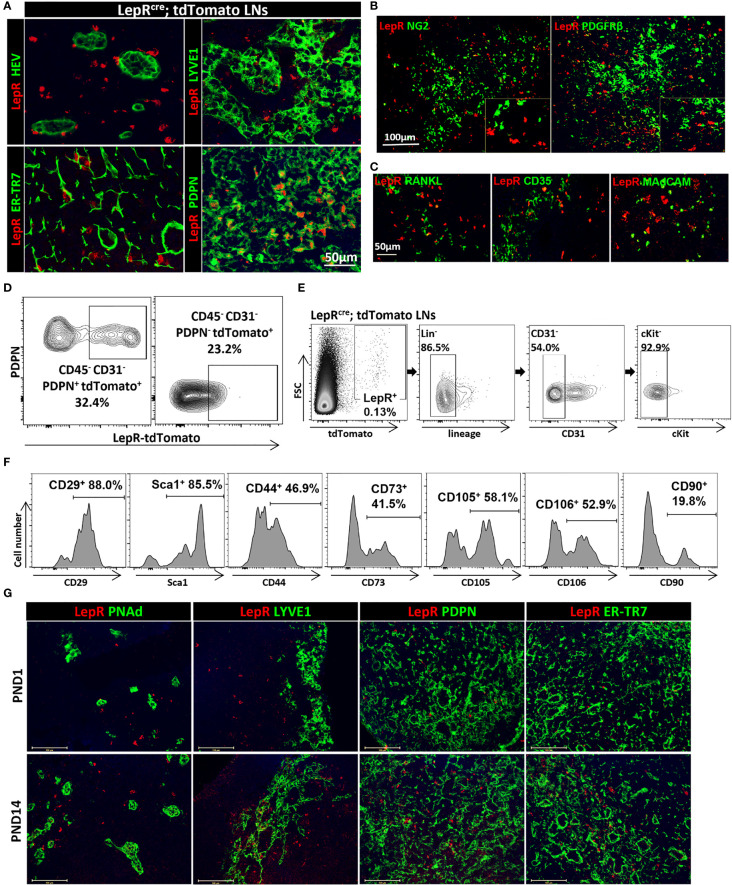
**(A)** Fluorescence micrographs show circumferential distribution of LepR^+^ cells (red) around HEVs and lymphatics in Lepr^cre^;tdTomato mice LNs. LepR (red) colocalized with PDPN and ER-TR7. Scale bar, 50μm. **(B)** Fluorescence micrographs of LNs from Lepr^cre^;tdTomato mice showed no colocalization of LepR (red) with NG2 and PDGFRβ. Scale bar, 100μm. **(C)** Fluorescence micrographs of Lepr^cre^;tdTomato mice LNs showed no colocalization of LepR (red) with RANKL, CD35 and MAdCAM. Scale bar, 50μm. **(D)** LepR expression in CD45^-^PDPN^+^CD31^-^(FRCs) and CD45^-^PDPN^-^CD31^-^(DN) populations of LNs were evaluated by flow cytometry. Data are representative of three independent experiments (n=3). **(E)** Gating strategy to exclude hematopoietic and endothelial cells for LepR^+^ cells in LepR^cre^;tdTomato LNs. **(F)** The percentages of LepR^+^ cells of LNs in stromal marker panel Sca-1, CD29, CD90, CD44, CD73, CD105 and CD106 were evaluated based on the gating strategy in **(E)**. Data are representative of three independent experiments (n=3). **(G)** Fluorescence micrographs of PND1 and PND14 LNs showed location of LepR^+^ cells (red) in relation to HEVs, lymphatics, PDPN and ER-TR7. Scale bar, 100μm.

We sought initially to identify the location of the LepR^+^ cells in mouse axillary LNs. LepR^Cre^;tdTomato mice were generated by crossing LepR^cre^ mice with Cre-inducible Rosa26-driven tdTomato mice. No tdTomato signal was found in the LNs from Rosa26-tdTomato mice without LepR-Cre (**Figure S1**). Sections of LepR^Cre^;tdTomato mouse LNs were stained with antibodies to FRC markers PDPN and ER-TR7, as well as to HEVs (PNAd) and lymphatic vessels (Lyve-1). **Figure 1A** shows that LepR^+^ cells (tdTomato^+^) were present in a circumferential pattern around HEVs (PNAd^+^). To fully characterize these LepR^+^ cells around HEVs, we used the pericyte markers NG2 and PDGFRβ to stain the LepR^Cre^;tdTomato LNs (**Figure 1B**). However, LepR^+^ cells did not express NG2 or PDGFRβ (**Figure 1B**). Next, we investigated the distribution of LepR^+^ cells within the interstitium of the LNs. We assessed the expression of LepR on PDPN^+^ FRCs and the ECM within the LNs using ER-TR7 antibody. We found that the many PDPN^+^ cells expressed LepR in LepR^Cre^;tdTomato mice (**Figure 1A**). ER-TR7 antigen (green) also colocalized partially with LepR (red) (**Figure 1A**). LepR^+^ cells were also found mostly adjacent to the lymphatic vessels, and fewer cells co-expressed Lyve-1 and LepR (**Figure 1A**). We also examined LepR expression by MRCs and FDCs. LepR^+^ cells did not express RANKL (MRCs), CD35 (FDCs), or MAdCAM (MRCs and FDCs) (**Figure 1C**). We performed flow cytometric (FACS) analysis of cells harvested from LepR^Cre^;tdTomato LNs. The results indicated that ~ 32% of CD45^-^PDPN^+^CD31^-^ FRCs expressed LepR (tdTomato positive, n=3), and ~23% of CD45^-^PDPN^-^CD31^-^ double negative cells (DNs) expressed LepR (**Figure 1D**). To examine the cellular characteristics of LepR^+^ cells with the LN further, we analyzed the expression of stromal markers on LepR^+^ cells from LepR^Cre^;tdTomato LNs. LepR^+^Lin^-^CD31^-^cKit^-^ (**Figure 1E**) cells from LepR^Cre^;tdTomato LNs were analyzed for the expression of stromal markers, which including FRCs. Our data (**Figure 1F**) showed co-expressionwith the stromal cell markers Sca1, CD29, CD90, CD44, CD73, CD105, and CD106 on LepR^+^ cells (85.5%, 88.0%, 19.8%, 46.9%, 41.5%, 58.1% and 52.9%, respectively).

Next, we examined the development of LepR^+^ cells in the postnatal (PND) period by staining sections of LNs retrieved on PND day 1 (PND1) and day 14 (PND14) from LepR^Cre^;tdTomato mice. As shown in **Figure 1G**, much fewer LepR^+^ cells were found in the PND1 LNs as compared to the PND14 LNs. LepR^+^ cells in these young mice also colocalized with the FRC markers PDPN and ER-TR7, a similar distribution as in the LNs of adult mice (**Figure 1G**).

### LN Abnormalities in LepR-Deficient Mice (db/db Mice)

**Figure 2:**
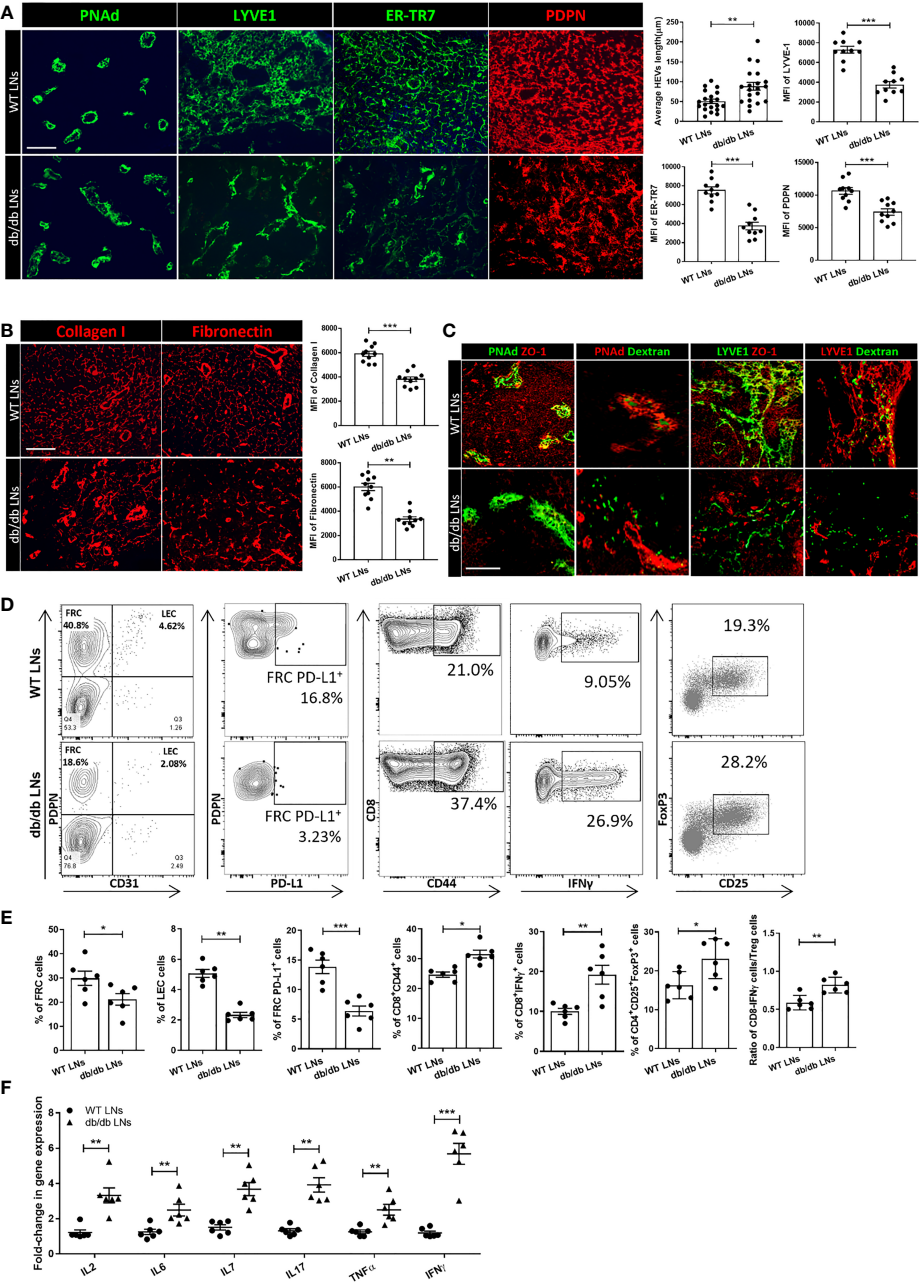
**(A)** Immunofluorescence staining showed differences in the appearance of ER-TR7^+^ fibers, PDPN+ cells, Lyve-1^+^ lymphatics, and HEVs between the LNs of WT mice and db/db mice. Scale bar, 50μm. Images are representative of five independent experiments (n=5). Quantification data from two independent experiments with five mice/group (n=5) are summarized in bar chart. All the data are presented as mean ± SEM, Student’s t test, **p < 0.05, ***p < 0.001. **(B)** Immunofluorescence staining showed differences between collagen I and fibronectin fibers in the LNs of db/db mice compared with WT mice. Scale bar, 50μm. Images are representative of five independent experiments (n=5). Quantification data from two independent experiments with five mice/group (n=5) are summarized in bar chart. All the data are presented as mean ± SEM, Student’s t test, **p < 0.05, ***p < 0.001. **(C)** Dextran injections were used to assess HEV and Lyve-1^+^ lymphatic vessel integrity. ZO-1 staining was used to assess the integrity of HEVs and lymphatic structures. Scale bar, 50μm. Images are representative of five independent experiments (n=5). **(D, E)** Flow cytometry profiling of WT and db/db mouse LN cell populations. Changes in the surface expression of PD-L1 was assessed in CD45^-^PDPN^+^CD31^-^ FRCs, CD44 expression was assessed in CD8^+^ T cells, production of IFNγ was assessed in CD8^+^ T cells, and CD4^+^CD25^+^FOXP3^+^ regulatory T cell populations were determined *via* intracellular staining followed by FACS analysis. Numbers in quadrants indicate cell percentages. Data from two independent experiments with six mice/group (n=6) are summarized in bar chart. All the data are presented as mean ± SEM, Student’s t test, *p < 0.05, **p < 0.01, ***p < 0.001. **(F)** Quantitative RT-PCR analysis was performed to examine inflammatory response-related gene expression in WT and db/db LNs. Data from two independent experiments with six mice/group (n=6). All the data are presented as mean ± SEM, Student’s t test, **p < 0.01, ***p < 0.001.

Next, we examined microanatomical changes within the LNs of db/db mice. As shown in **Figure 2A**, the HEVs in the db/db LNs were expanded and elongated, and the walls were thinner in comparison to WT LNs. To visualize the HEVs more precisely, we performed 3D imaging of solvent-cleared organs (iDISCO) of WT LNs and db/db LNs, indicating a marked expansion of db/db HEVs, as compared with WT LNs (**Figure S2A**). We also investigated the changes in the Lyve-1^+^ lymphatic plexus. In contrast to the linear lymphatics of the WT LNs, those of the db/db LNs were discontinuous (**Figure 2A**). We examined subsequently the ER-TR7^+^ reticular fibers and PDPN expression in db/db mice, as compared with WT mice. The structure of the ER-TR7^+^ reticular fibers and the microarchitecture formed by PDPN^+^ cells in the LNs of the db/db mice showed dense clusters that lacked the thin homogenous distribution of matrix seen in the WT LNs (**Figure 2A**). Under physiological conditions, FRCs secrete collagen fibers that form an elaborate ECM network. We stained the LNs for collagen I and fibronectin, and we found that the architectural framework was disorganized into dense clusters of fibers in the db/db LNs (**Figure 2B**). Next, we determined whether impaired barrier function leads to increased permeability of HEVs and lymphatics. We injected dextran tagged with fluorescein (dextran-FITC) intravenously or subcutaneously to analyze the permeability of HEV and lymphatics by IF staining. 2000kDa Dextran-FITC was restricted to the lumens of the HEVs and lymphatics of the WT LNs, while it leaked from the HEVs and lymphatics into the tissue in the db/db LNs (**Figure 2C**). Interestingly, the expression of the tight junction protein zonula occludens-1 (ZO-1) was lower in the lymphatics and HEVs of the db/db LNs (**Figure 2C**). We also assessed ZO-1 and two other tight junction markers–claudin5 and occludin–with respect to mRNA levels by RT-PCR, and all were reduced in the db/db LNs (**Figure S2B**). Taken together, these data indicate that LepR signaling plays a critical role in maintaining the integrity of HEVs and lymphatic vessels in murine LNs.

We examined the FRC populations in db/db and WT LNs by flow cytometric analysis, which revealed that the proportion of FRCs and lymphatic endothelial cells (LECs) in the LNs of db/db mice were significantly lower in comparison to those in WT mice (21.13 ± 5.910% vs 29.83 ± 7.282%, 2.335 ± 0.6383% vs 5.06 ± 0.4224%, respectively, n=6/group) (**Figures 2D, E**). PD-L1 is an anti-inflammatory mediator that is expressed constitutively by FRCs (28). Flow cytometric analysis also indicated a downregulation of PD-L1 expression in the FRCs of the db/db LNs, as compared to the WT LNs (6.372 ± 2.06% vs 13.85 ± 2.77%, n =6/group, respectively). Then, we analyzed the phenotypes of the T cell population of the LNs in the db/db mice and compared them to those in the WT LNs. We noted an upregulation of activation marker CD44 (31.57 ± 3.173% vs 24.67 + 2.16%, n =6/group) and pro-inflammatory cytokine IFNγ (19.22 ± 5.852% vs 10.02 ± 1.89%, n =6/group) in CD8^+^ T cells of db/db mice. A higher percentage of the CD4^+^CD25^+^FOXP3^+^ regulatory T cell (Treg) population (23.86 ± 4.716% vs 17.23 ± 2.018%, n =6/group) was also noted in the LNs of the db/db mice as compared to the LNs of the WT mice (**Figures 2D, E**). Flow cytometric analysis demonstrated that IFNγ was upregulated by CD8^+^ T cells in the db/db LNs, as compared to the WT LNs. Although the Treg population was also increased in db/db LNs, the ratio between the CD8^+^IFNγ^+^ T cells and Treg populations was higher in the db/db mice than the WT mice, indicating a shift towards a pro-inflammatory milieu in the LNs of the db/db mice. RT-PCR was performed to confirm the expression of inflammatory cytokines that play important roles in T2D in db/db mice LNs. As shown in **Figure 2F**, gene expression levels of IL-2, IL-6, IL-7, IL-17, TNFα, and IFNγ were higher in db/db LNs than WT LNs.

### Leptin Regulates the Survival and Function of Cultured FRCs

**Figure 3:**
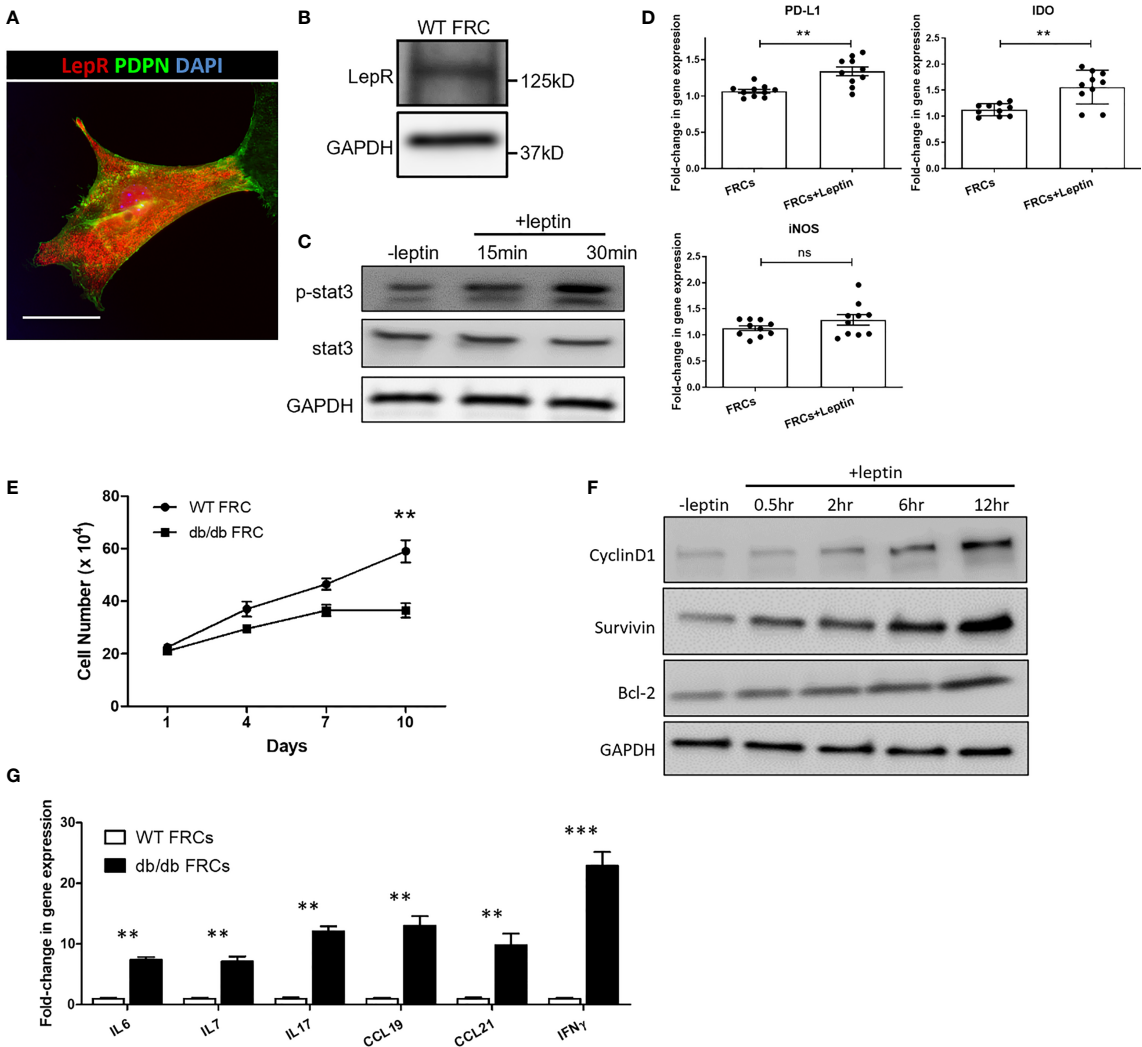
**(A)** Fluorescence micrographs show the expression of LepR (red) in FRCs from Leprcre;tdTomato mice LNs. Scale bar, 10μm. **(B)** The expression of LepR in FRCs was confirmed by Western blot. **(C)** Immunoblot analysis of pSTAT3 (Y705) and STAT3 in FRCs exposed to 100 ng/ml leptin for 15 min and 30 min. GAPDH was used as a loading control. **(D)** Quantitative RT-PCR analysis was performed on FRCs RNA to examine the expression of iNOS, IDO, and PD-L1 in untreated FRCs and FRCs exposed to 100 ng/ml of leptin for 24 hrs. The data are representative of two independent experiments with five mice/group. All the data are presented as mean ± SEM, Student’s t test, **p < 0.01, n.s., not significant. **(E)** Cell growth curve. Proliferation rate of FRCs from WT and db/db mice, as measured by cell count. The values are expressed as three independent measurements. All the data are presented as mean ± SEM, Student’s t test, **p < 0.01. **(F)** Immunoblotting analysis of the cell survival markers cyclinD1, survivin, and Bcl-2 in FRCs exposed to 100 ng/ml leptin for 0.5 hr, 2 hr, 6 hr, and 12 hr. GAPDH was used as a loading control. **(G)** Quantitative RT-PCR analysis was performed to examine inflammatory response-related gene expression in WT and db/db FRCs. The data are representative of three independent experiments. All the data are presented as mean ± SEM, Student’s t test, **p < 0.01, ***p < 0.001.

LepR^+^ FRCs were sorted and cultured from LNs, as previously described (29). First, we confirmed the presence of LepR in FRCs from LepR^Cre^;tdTomato mice LNs by IF staining and Western blot (**Figures 3A, B**). Leptin regulates cellular homeostasis by activating multiple intracellular signaling cascades, including the JAK/STAT pathway *via* LepR (30). As shown in **Figure 3C**, immunoblotting analysis demonstrated that leptin increased the phosphorylation of STAT3 (Tyr705) in FRCs (**Figure 3C**). To determine whether leptin induces the production of anti-inflammatory molecules, we examined the expression of PD-L1, iNOS and IDO at the transcriptional level in FRCs treated with leptin. As shown in **Figure 3D**, quantitative real-time PCR indicated that exposure to leptin enhanced the expression of PD-L1 and IDO mRNA with no difference in the expression of iNOS.

To further investigate differences in the activities of WT and db/db FRCs, we cultured and assessed proliferation of WT and db/db FRCs *in vitro*. The proliferation of db/db FRCs stalled after Day 7, as the WT FRCs continued to grow (**Figure 3E**). We exposed WT FRCs to 100 ng/ml of leptin for 0.5 hr, 2 hr, 6 hr, and 12 hr. The cell survival markers cyclinD1, survivin, and bcl-2 were activated more highly after 6 hr in the leptin-treated FRCs in comparison to the group that did not receive leptin (**Figure 3F**). These data indicated that LepR signaling plays an important role in the survival of FRCs, as substantiated by a reduction of the FRC population in db/db mice. In addition, FRCs isolated from db/db mice demonstrated higher levels of expression of CCL19 and CCL21 genes than WT FRCs by qPCR (**Figure 3G**). CCL19 and CCL21 are responsible for recruiting T cells and dendritic cells *via* CCR7 (31). IL-7 is known for its critical role in the development and homeostatic expansion of T cells in mice and contributes to the survival of naïve T cells (32, 33). The mRNA levels of IL-7 were higher in the db/db FRCs (**Figure 3G**). IL-6, an inflammatory cytokine, also was present at a higher level in db/db FRCs in comparison to WT FRCs (**Figure 3G**).

### Leptin Treatment Restores the Structure and Function of LNs in ob/ob Mice

**Figure 4:**
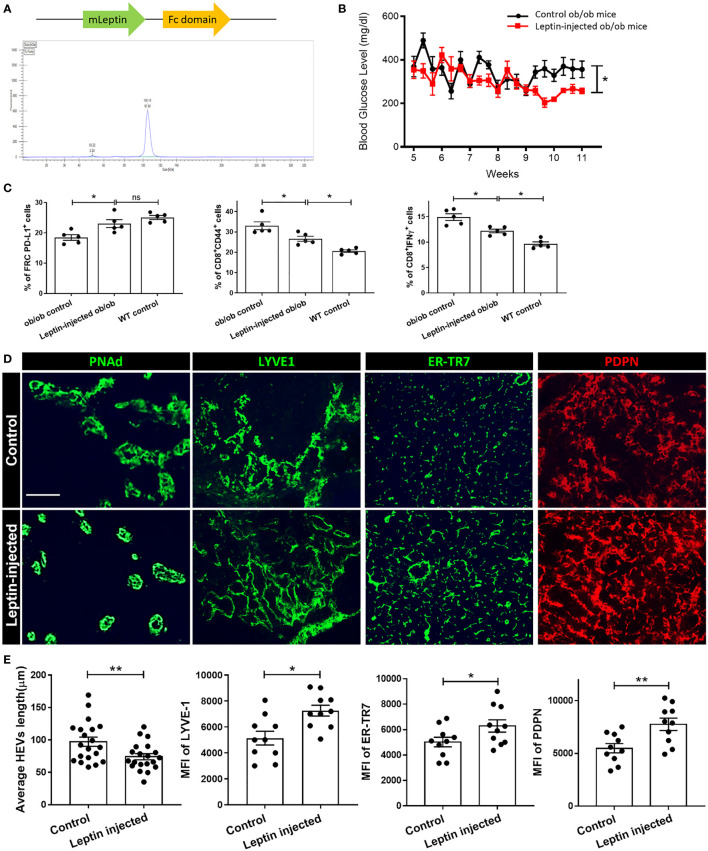
**(A)** Schematic of Leptin-Fc recombinant protein and HPLC purification curve. **(B)** Metabolic phenotype of ob/ob mice that were injected with 0.25 mg leptin/kg body weight or Fc for 6 weeks, and blood glucose was measured. **(C)** Flow cytometry profiling of cell population changes in the LNs of ob/ob control, leptin-treated ob/ob, and WT control mice. Changes in the surface expression of PD-L1 was assessed in CD45^-^PDPN^+^CD31^-^ FRCs, CD44 expression was assessed in CD8^+^ T cells, production of IFNγ was assessed in CD8^+^ T cells. Data from two independent experiments with five mice/group (n=5) are summarized in bar chart. All the data are presented as mean ± SEM, Student’s t test, *p < 0.05, n.s., not significant. **(D)** Fluorescence micrographs show restoration of microarchitecture in leptin-treated ob/ob mouse LNs in comparison to control LNs, as demonstrated by expression of PDPN, ER-TR7, HEV and Lyve-1. Scale bar, 50μm. Images are representative of five independent experiments from five mice/group (n=5). **(E)** Quantification data from two independent experiments with five mice/group (n=5) are summarized in bar chart. All the data are presented as mean ± SEM, Student’s t test, *p < 0.05, **p < 0.01.

We generated and purified a leptin-Fc fusion recombinant protein (**Figure 4A**). Ob/ob mice were injected intraperitoneally each day with recombinant leptin protein 0.25 mg/kg for 6 weeks (from 5 weeks through 11 weeks of age) and compared to a control group of ob/ob mice that received daily PBS injections. Exogenous administration of leptin decreased plasma glucose levels and increased the expression of PD-L1 by FRCs in the treated group, as compared to the control groups (**Figures 4B, C**, respectively). The percentages of activated CD8^+^ T cells (CD8^+^CD44^+^) and CD8^+^ IFNγ^+^ T cells in the LNs of the leptin-treated group were significantly lower than the untreated ob/ob control group.

As shown in **Figures 4D, E**, and **S3B**, treatment with leptin-Fc fusion protein restored the PDPN^+^ cells and ER-TR7^+^ fiber network to a more organized and uniformly distributed configuration in the treated group, as compared to the controls. Consistent with these findings, the typical structure of the HEVs and the Lyve-1^+^ lymphatics was restored in the LNs from the leptin-injected mice (**Figures 4D, E**). We also examined the integrity of the HEVs and lymphatics by dextran-FITC injection. Although leakiness was observed in these vessels, the quantification analysis showed that the vessels in the LNs of the leptin-treated group were significantly less leaky than the controls (**Figure S3A**). Examination of HEVs and Lyve-1^+^ LECs provided evidence of a more organized support structure composed of ER-TR7^+^ fibers secreted by FRCs that surrounded the HEVs and Lyve-1^+^ lymphatic vessels in the LNs of the leptin-treated mice in comparison to the control mice.

### Assessment of LNs in CCL19^cre^;LepR^fl^ Conditional Knock Out Mice

**Figure 5:**
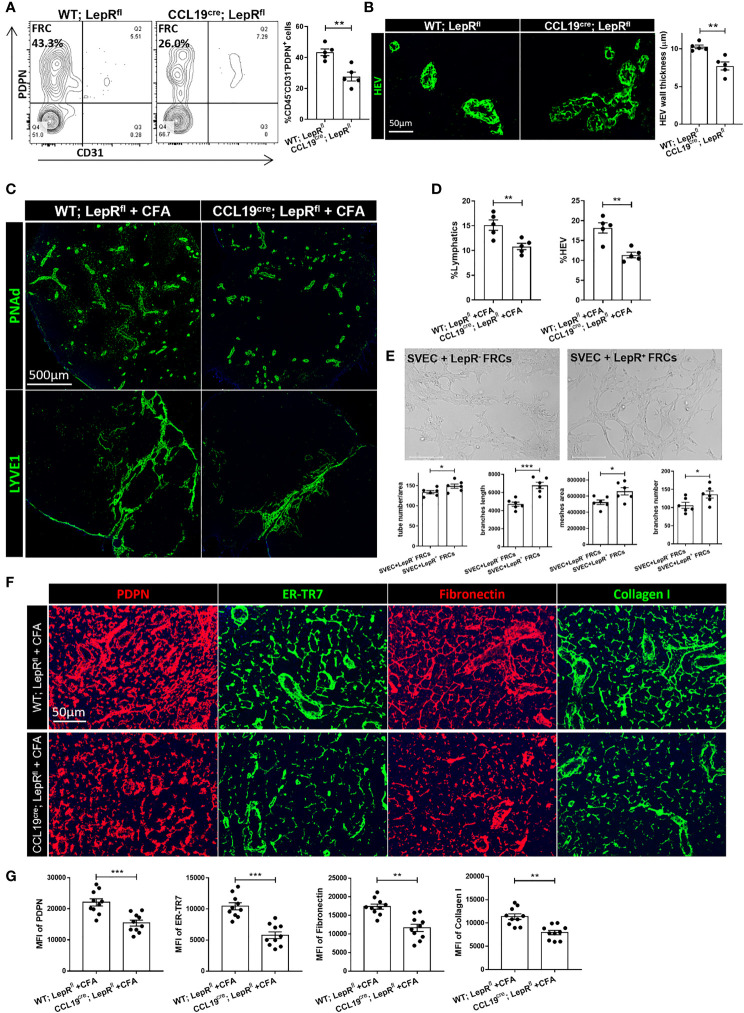
**(A)** Changes in CD45^-^PDPN^+^CD31^-^FRC populations between WT;LepR^fl^ and CCL19^cre^;LepR^fl^ mouse LNs, as determined by flow cytometric analysis. Data from two independent experiments with five mice/group (n=5) are summarized in bar chart. All the data are presented as mean ± SEM, Student’s t test, **p < 0.01. **(B)** Immunofluorescence staining shows thinner HEV walls in CCL19^cre^;LepR^fl^ mouse LNs than WT;LepR^fl^ mouse LNs. Quantification data of HEV wall thickness are summarized in bar chart. Data from two independent experiments with five mice/group (n=5) are summarized in bar chart. All the data are presented as mean ± SEM, Student’s t test, **p < 0.01. **(C)** Fluorescence micrographs showed HEV and lymphatic expansion induced by CFA in WT;LepR^fl^ and CCL19^cre^;LepR^fl^ mouse LNs. Scale bar, 500μm. Images are representative of two independent experiments from five mice/group (n=5). **(D)** Quantification data from two independent experiments with five mice/group (n=5) are summarized in bar chart. All the data are presented as mean ± SEM, Student’s t test, **p < 0.01. **(E)** Tube formation capacity of SVEC4-10 upon leptin pretreated LepR^+^ FRCs supernatant, comparing with LepR^-^ FRCs *in vitro*. Capillary-like structures within the Matrigel layer were photographed after 48 hrs. Scale bar, 200μm. The area of tube-like formation, wall thickness and number of SVEC4-10 were evaluated by ImageJ software. Quantification data are presented as the mean ± SEM (n=6), All the data are presented as mean ± SEM, Student’s t test, *p < 0.05, ***p < 0.001. **(F)** Immunofluorescence staining showed the microarchitecture changes of ER-TR7, PDPN, collagen I and fibronectin in WT;LepR^fl^ and CCL19^cre^;LepR^fl^ mice LNs with CFA stimulation. Scale bar, 50μm. Images are representative of two independent experiments from five mice/group (n=5). **(G)** Quantification data from two independent experiments with five mice/group (n=5) are summarized in bar chart. All the data are presented as mean ± SEM, Student’s t test, **p < 0.01, ***p < 0.001.

To characterize the effects of leptin on the phenotype of the LNs more deeply, we generated mice with conditional ablation of LepR, using an FRC-selective Cre (CCL19^cre^;LepR^fl^) (34, 35). First, we examined the specificity and efficiency of deletion of the target cell population in CCL19^cre^;LepR^fl^ mice. LepR-expressing FRCs were shown to be reduced in the LNs of these mice, as compared to other cell types (**Figure S4A**). The percentage of resident FRCs in the stromal cell population in the LNs from CCL19^cre^;LepR^fl^ mice was lower than the WT;LepR^fl^ control mice (26.0% vs 43.3%, **Figure 5A**). HEVs of CCL19^cre^;LepR^fl^ mice possessed thinner walls, as compared with the control mice (**Figure 5B**). PDPN expression was less dense, and the ER-TR7^+^, fibronectin, and collagen I fiber network was thinner in the CCL19^cre^;LepR^fl^ mice (**Figure S4B, C**). Nonetheless, the populations of IL6^+^, IL7^+^, CD8^+^IFNγ^+^, and CD4^+^CD25^+^FoxP3^+^ cells were not significantly different between the WT;LepR^fl^ control and CCL19^cre^;LepR^fl^ mice (**Figure S4D**).

Then, we immunized these mice with Complete Freund’s Adjuvant (CFA) to investigate the pattern of acute immune responses. Seven days after subcutaneous CFA injection, the draining LNs (DLNs) were significantly smaller in the CCL19^cre^;LepR^fl^ mice than the WT;LepR^fl^ control mice (**Figure S4E**). As shown in **Figures 5C, D**, HEVs and lymphatics were significantly more expanded in the DLNs of the control groups, as compared to the DLNs of CCL19^cre^;LepR^fl^ mice. No significant leak of dextran-FITC into the DLNs of either the control or CCL19^cre^;LepR^fl^ mice was found (data was not shown). Immunofluorescence staining revealed that PDPN, ER-TR7, fibronectin, and collagen I were significantly more upregulated in the DLNs of the control mice than the CCL19^cre^;LepR^fl^ mice after CFA stimulation (**Figures 5F, G**). We also measured the percentages of PD-L1^+^ FRCs and CD8^+^IFNγ^+^ T cells in both groups, and no significant difference was found (**Figure S4F**). To investigate more thoroughly the importance of LepR cells in supporting the expansion of lymphatics, we sorted LepR^-^ and LepR^+^ FRCs and co-cultured them with the murine LEC cell line SVEC4-10. As shown in **Figure 5E**, LepR^+^ FRCs promoted tube formation and branch elongation of SVEC cells *in vitro.*

Next, we performed CFA + OVA immunization to assess the cell-mediated and humoral immune responses in CCL19^Cre^;LepR^fl^ and control mice. Fourteen days after subcutaneous immunization, DLNs were collected for flow cytometry. No difference in the percentages of T cells, follicular T cells (CD4^+^CXCR5^+^ICOS^+^), Tfh cells (CD4^+^CXCR5^+^ICOS^+^FoxP3^-^), and proliferating Tfh cells (CD4^+^CXCR5^+^ICOS^+^FoxP3^-^Ki67^+^) was seen (**Figure S4G**). Moreover, no difference was observed in total B cells, plasma cells, and antibody concentration between the WT;LepR^fl^ control and CCL19^cre^;LepR^fl^ mice (**Figure S4H**).

## Discussion

LNs are crucial to the maintenance of immune responses, both at the steady-state and following activation (36). Takeuchi et al. have reported that a fibroblastic stromal cells (FSC) subset expressing LepR in the medullary cord, typically consisting of lymphatics, was identified using a polyclonal antibody against LepR (37). LepR has different isoforms that could be recognized by the LepR antibody. obRb is the sole functional form that can transduce an extracellular signal to the cytoplasm (38). In this study, we used LepR^Cre^;tdTomato reporter mice and found that LepR-expressing cells are also present within the vicinity of the HEVs and the lymphatics. Quantification of the stromal cells showed that ~30% of the PDPN^+^CD31^-^ FRCs and ~20% of the double negative cell population express LepR, which could be representative of stromal progenitor cells in the LNs. LepR^+^ cells expressed a high percentage of mesodermal markers, including Sca1, CD29, CD90, CD44, CD73, CD105, and CD106. However, MRCs and FDCs lacked expression of LepR. Morrison’s group showed previously that LepR^+^ stromal cells are the important cellular component of hematopoietic stem cells (39). These cells share many features of mesenchymal stem cells (MSCs), which are multipotent stromal cells with the capacity to differentiate into various mesodermal lineages (38). Lineage tracing studies can help to define the origin of LepR^+^ cells in the LN and the trafficking dynamics of these cells to the LN.

The stroma of LNs plays a pivotal role in supporting their structure and function (40). Many advances have been made in characterizing stromal cells, according to their location within discrete anatomical areas of the LNs and with specific markers (41, 42). FRCs not only build the scaffolding of the LN; they also support the integrity of its vasculature (7, 40, 43). The ECM proteins collagen I, fibronectin, and ER-TR7, as well as the PDPN^+^ cells were thinner and more disorganized in db/db mice. Moreover, the permeability of HEVs and lymphatic vessels was increased, as both were leaky in db/db LNs. Taken together, these data show that leptin signaling is required for the proper functioning of the FRC network in its maintenance of LN stroma as well as the integrity of HEVs and lymphatics in LNs.

FRCs propagate immune responses through production of ECM and homing of immune cells, but they also can downregulate inflammatory responses by expressing immunoregulatory molecules (44). FRC-derived chemokines and cytokines recruit naive T cells and ensure T cell survival within LNs (45). FRCs can also induce deletional T cell tolerance directly and restrict the expansion of newly activated T cells (8). Indeed, expression of immunosuppressive molecules, such as PD-L1 and IDO, represents a key feature of FRCs. The PD-L1 pathway delivers inhibitory signals that regulate peripheral T-cell tolerance (46, 47). Our data show that the LNs of db/db mice contained a lower percentage of PD-L1-expressing FRCs, as compared to WT mice. The ratio of CD8^+^ IFNγ^+^ T cells to Treg cells was also much higher in db/db LNs. These findings indicate a heightened pro-inflammatory milieu in the LNs of db/db and suggest that LepR^+^ FRCs may have immunoregulatory features. Treatment of FRCs cultured *in vitro* with leptin resulted in the production of the anti-inflammatory molecules PD-L1 and IDO. Therefore, leptin signaling may promote the population of immunoregulatory FRCs that in turn determine the inflammatory milieu of the LNs. These data derived from the LNs of db/db mice open a new avenue of research to understand the dysregulated inflammatory responses in the LNs of T2D patients.

Leptin has been shown to regulate lymphatic homeostasis, such as tube formation and cell proliferation (48, 49). Previous studies demonstrated that leptin increases proliferation and reduces apoptosis of human dermal LECs (50, 51). Here, we showed that leptin activated the JAK/STAT pathway downstream of LepR, which then increased survival-related protein cyclinD1, survivin, and Bcl-2 expression. In contrast, db/db FRCs displayed growth deficiency.

We also tested the immunoregulatory effect of leptin on the LN stroma by generating a leptin fusion protein, which we administered to leptin-deficient ob/ob mice. The treatment with leptin fusion protein improved the structural integrity of the HEVs and the Lyve-1^+^ lymphatic plexus as compared to the controls. The PDPN^+^ and ER-TR7^+^ fiber network in leptin-injected LNs was more contiguous, thicker, and more uniformly distributed. Leptin fusion protein also increased the population of PD-L1-expressing FRCs and reduced the population of activated T cells in the LNs. These data suggest that future studies should be directed towards the delivery of leptin agents to the LNs. Along with others, we have previously shown that targeted delivery of therapeutics to LNs represents an innovative approach to suppress inflammatory conditions, such as transplant rejection (52–54).

Global LepR-deficient db/db mice are obese and suffer a range of systemic metabolic issues (55, 56), which could also affect the LNs. To support our findings regarding the importance of LepR^+^ FRCs in the LNs, we generated a conditional ablation of LepR using an FRC-selective Cre recombinase (CCL19^cre^;LepR^fl^). CCL19^cre^;LepR^fl^ mice contained fewer FRCs in the LNs as well as thinner HEVs. The ECM of CCL19^cre^;LepR^fl^ LNs was disconnected from the network of PDPN^+^ cells and ER-TR7^+^ fibers, and these LNs contained a lower density of collagen I and fibronectin fibers. Challenge of CCL19^cre^;LepR^fl^ mice with the inflammatory stimulus CFA showed a lower level of vascular and stromal accommodation as compared to the controls. Co-culturing LepR^+^ FRCs with the lymphatic cell line SVEC4-10 induced expansion of the SVEC4-10 cells, as they formed tubes on the bottom of the flask.

The clinical impact of these data lies in the potential importance of LN dysregulation and its implications in the pathogenesis of metabolic syndromes, such as T2D (15, 57). T2D affects millions of patients worldwide and is characterized by heightened inflammatory responses (58–60). While future studies are required to define mechanisms by which FRCs control the development or progression of T2D at the level of the pancreas or in peripheral organs, our data strongly suggest that this line of research could yield highly clinically applicable findings. These future studies could also lay the groundwork for new LN-targeted therapies to treat the metabolic syndrome.

## Materials and Methods

### Mice

BKS.Cg-Lepr^db^/J (JAX#000642, Homozygous, db/db), BKS.Cg-Dock7^m+/+^/J (JAX#000642, Homozygous, WT), B6.Cg-Lep^ob^/J (JAX#000632, Homozygous, ob/ob) B6.129(Cg)-Lepr^tm2(cre)Rck^/J (JAX#008320, Homozygous, LepR^cre^), B6.129P2-Lepr^tm1Rck^/J (JAX#008327, Homozygous, lepR^fl/fl^) and B6.Cg-Gt(ROSA)26Sor^tm14(CAG-tdTomato)Hze^/J (JAX#007914, Homozygous, Rosa26-CAG-loxp-stop-loxp-tdTomato) mice were obtained from the Jackson Laboratory. CCL19^Cre^ [Tg(Ccl19-cre)489Biat] mice were a gift from Shannon Turley at Genentech, South San Francisco, California, USA. All animal experiments and methods were performed in accordance with the relevant guidelines and regulations approved by the Institutional Animal Care and Use Committee of Brigham and Women’s Hospital, Boston, MA.

### Immunohistochemistry

Fresh LNs were embedded in tissue-freezing medium. Cryostat sections (8 μm thick) were cut for imaging by fluorescence confocal microscopy. The following primary Abs were used for tissue staining: anti-HEV MECA79 (sc-19602, SCBT), anti-Lyve-1 (ab14917, Abcam), anti-PDPN (AF3244, R&D Systems), anti-ER-TR7 (sc-73355, SCBT), anti-CD11b (101202, BioLegend), anti-PDGFRβ (136005, Biolegend), anti-NG2 (ab129051, Abcam), anti-RANKL (510002 Biolegend), anti-CD35 (NBP2-52667, Novus Biologicals), anti-MAdCAM (16-5997-85,eBioscience), anti-ZO-1 (61-7300, Invitrogen), anti-Collagen I (ab34710, Abcam), anti-Fibronectin (ab45688, Abcam), anti-LepR (L9536, Sigma-Aldrich). The following secondary Abs were used: Alexa Fluor 488-conjugated anti-rabbit IgG, Alexa Fluor 594-conjugated anti-rabbit IgG, Alexa Fluor 488-conjugated anti-rat IgG, Alexa Fluor 594-conjugated anti-rat IgG, Alexa Fluor 488-conjugated anti-goat IgG, and Alexa Fluor 594-conjugated anti-goat IgG (Jackson ImmunoResearch). The stained tissue sections were imaged using EVOS FL Auto 2 Imaging System (Thermo Fisher Scientific). For the quantification of images, all images were automatically processed using ImageJ (NIH) and split into RGB channels. Auto threshold was used to convert intensity values of the immunofluorescent stain into numeric data. DAPI (VECTASHIELD, Vector Laboratories) was used to stain the cell nuclei.

### Flow Cytometry Analysis

At given experimental time points, mice were killed, and LNs were isolated for flow cytometry. Single-cell suspensions were prepared using an enzyme mixture, comprised of RPMI-1640 medium containing 0.8 mg/ml Dispase, 0.2 mg/ml Collagenase P (both from Roche), and 0.1 mg/ml DNase I (Invitrogen). LNs were incubated at 37°C and gently mixed using a pipette at 5 to 15 min intervals to ensure the proper dissociation of cells. After complete dissociation, the cell mixture was filtered through a 40-μm cell strainer, counted, and used for surface and intracellular staining. Then, cells were resuspended in FACS buffer (PBS containing 2% FBS and 5 mM EDTA). Cells were incubated for 30 min with antibodies against the indicated markers: APC anti-CD45, PerCP anti-CD31, PE/Cy7 anti-PDPN, PB anti-lineage cocktail, APC anti-cKit, PE/Cy7 anti-Sca1, FITC anti-CD29, Brilliant Violet 510 anti-CD90, FITC anti-CD44, PerCP anti-CD73, APC anti-CD105, PE anti-CD106, Brilliant Violet 510 anti-CD4, PE anti-CD25, PE/Cy7 anti-CD44, APC/Cy7 anti-CD8, and PE/Cy7 anti-PD-L1 (Biolegend). The cells were permeabilized using the eBioscience Intracellular Fixation and Permeabilization Buffer Set (Thermo Fisher Scientific) for 30 min at 4°C. Then they were incubated with the following intracellular antibodies: PerCP/Cy5.5 anti-FoxP3, APC anti-IFNγ (Biolegend). Cells were washed once with Permeabilization Buffer and fixed in FACS buffer containing 1% formalin. For B and T cell analyses after immunization, cells were stained with APC/Cy7 or Brilliant Violet 510 anti-CD4, APC/Cy7 or Brilliant Violet 510 anti-CD19, Pacific Blue anti-CD38, FITC T and B cell activation marker (GL-7), PE/Cy7 anti-Fas, APC anti-CD138, biotin anti-CXCR5, and Brilliant Violet 421 Streptavidin, PE anti-ICOS, PE/Cy7 anti-PD-1, and intracellularly stained with Alexa Fluor 488, anti-Foxp3, and Alexa Fluor 647 anti-Ki67. Flow cytometry was performed using a BD FACSCantoTM II flow cytometer (BD Biosciences) or Aurora (Cytek). Analysis of flow cytometry results was performed *via* FlowJo software (FlowJo LLC, Ashland, OR).

### Quantitative RT-PCR

RNA was isolated with TRIZOL (Invitrogen), and first strand cDNA was synthesized using 2 μg of RNA and High-Capacity Reverse Transcriptase (Invitrogen). RT-PCR was performed with SYBR Green PCR reagents on a Bio-Rad detection system. RNA levels were normalized to the level of GAPDH and calculated as delta-delta threshold cycle (ΔΔCT). Primers used for RT-PCR are listed as follows: GAPDH-F:AGCCACATCGCTCAGACAC; GAPDH-R:GCCCAATACGACCAAATCC; IL2-F:TGAGCAGGATGGAGAATTACAGG; IL2-R:GTCCAAGTTCATCTTCTAGGCAC; IL6-F:CTCTGGGAAATCGTGGAAAT; IL6-R:CCAGTTTGGTAGCATCCATC; IL7-F:TCTGCTGCCTGTCACATCATC; IL7-R:CCTTTGTATCATCACATACAT; IL17-F:AAGGCAGCAGCGATCATCC; IL17-R:GGAACGGTTGAGGTAGTCTGAG; CCL19-F:TGTGTTCACCACACTAAGGGG, CCL19-R:CCTTTGTTCTTGGCAGAAGACT; CCL21-F:CCCCTGGACCCAAGGCAGTGA; CCL21-R:TTGCCGGGATGGGACAGCCT; TNFα-F:ATGAGAAGTTCCCAAATGGC; TNFα-R:CTCCACTTGGTGGTTTGCTA; IFNγ-F:TTGAGGTCAACAACCCACAG; IFNγ-R:TCAGCAGCGACTCCTTTTC; iNOS-F:ACCTTGTTCAGCTACGCCTT; iNOS-R:CATTCCCAAATGTGCTTGTC; IDO-F:GTACATCACCATGGCGTATG; IDO-R:CGAGGAAGAAGCCCTTGTC; PDL1-F:GACCAGCTTTTGAAGGGAAATG; PDL1-R:CTGGTTGATTTTGCGGTATGG; Claudin5-F:CCTTCCTGGACCACAACATC; Claudin5-R:GCCGGTCAAGGTAACAAAGA; Occludin-F:CCTCCAATGGCAAAGTGAAT; Occludin-R:CTCCCCACCTGTCGTGTAGT; ZO1-F:CCACCTCTGTCCAGCTCTTC; ZO1-R:CACCGGAGTGATGGTTTTCT. All RT-PCR reactions were performed in triplicate.

### Immunoblotting

Whole lymph node cells or FRC lysates were measured using the Bradford assay. Equal amounts of protein were separated by SDS-PAGE and transferred to a PVDF membrane. The membranes were immunoblotted with the following specific antibodies: anti-LepR (Sigma), anti-pSTAT3 (Cell Signaling), anti-STAT3 (Cell Signaling), anti-GAPDH (Santa Cruz), anti-cyclinD1 (Cell Signaling), anti-survivin (Cell Signaling), anti-Bcl2 (Cell Signaling), anti-rabbit-HRP (Abcam), anti-mouse-HRP (Jackson ImmunoResearch), and anti-goat-HRP (Abcam) using standard protocols. The blots were developed with West Dura chemiluminescent substrates using a Bio-Rad ChemiDoc imaging system.

### Purification of Leptin-Fc Recombinant Protein

The full-length mouse leptin gene was constructed in a mammalian expression vector with Fc fusion tag. HEK293T cells were transiently transfected with the plasmid in the presence of *polyethylenimine* (PEI). Media supernatants were collected after 4 days and applied to protein G Sepharose 4 Fast Flow (GE Healthcare). Leptin-Fc recombinant protein was eluted with a low pH elution buffer.

### Treatment With Leptin-Fc Recombinant Protein

Either Leptin-Fc recombinant protein (0.25mg/kg) or Fc was injected intraperitoneally into B6.Cg-Lep^ob^/J (JAX#000632, Homozygous, ob/ob) every 4 days for 6 weeks. Blood glucose was checked three times per week and compared between groups.

### Treatment With CFA

100ul CFA was injected subcutaneously. LNs were examined at 7 days post-injection. For NP-OVA immunization, mice were injected subcutaneously in the flank with 100ug of NP-OVA in 100uL of CFA. Inguinal lymph nodes were collected for analysis 14 days after immunization.

### SVEC Cell Culture and Tube Formation

SVEC4-10 cells were cultured in complete DMEM (containing 10% fetal bovine serum). LepR^+^ FRCs were sorted and pretreated with leptin 100ng/ml. After 6 hours, supernatant was collected and incubated with SVEC cells in Matrigel (BD Biosciences) for 24 hours in 37°C. The capillary tube structures were observed, and representative images were captured with an EVOS FL Auto 2 Imaging System (Thermo Fisher Scientific). Tube area and wall thickness were quantified by ImageJ software (http://rsbweb.nih.gov/ij/; National Institutes of Health, Bethesda, MD). Briefly, three randomly selected fields of view were photographed in each treatment. Tube wall thickness was assessed by drawing a line along each tube and measuring the length of the line in pixels. The average of three fields was taken as the value for each treatment.

### Statistical Analysis

Statistical analysis was performed with Prism 5 (GraphPad Software). Data are presented as mean ± SEM. Statistical analysis was performed using the unpaired 2-tailed Student’s t test to determine differences between 2 groups and analysis of variance to compare data among groups. P values of less than 0.05 were considered statistically significant. Each experiment was repeated at least twice with similar results.

## Data Availability Statement

The original contributions presented in the study are included in the article/**Supplementary Material**. Further inquiries can be directed to the corresponding authors.

## Ethics Statement

The animal study was reviewed and approved by Institutional Animal Care and Use Committee of Brigham and Women’s Hospital, Harvard Medical School.

## Author Contributions

LJ designed and performed experiments, analyzed data, wrote the main text of the manuscript, and critically revised and finalized the manuscript. MY performed experiments. MU performed experiments, microsurgery, analyzed data, and wrote parts of the methods. CBC, JZ, SMN, XL and NB performed experiments. VK, PF, SRS, SGT, JSB and PTS edited the manuscript. RA designed the study, interpreted, analyzed data, and critically revised and finalized the manuscript. All authors contributed to the article and approved the submitted version.

## Funding

This work was supported in part by the National Institute of Allergy and Infectious Diseases and National Heart, Lung, and Blood Institute of the National Institutes of Health (NIH) under award numbers R01HL145813(RA), R01HL141815(RA), R01AI126596 (RA), R01AI156084 (RA), P01AI153003 (RA), and K24AI116925 (RA). This work was also supported by Hundred-Talent Youth Program (Chinese Academy of Sciences) under award numbers E1BDEDF6241.

## Conflict of Interest

The authors declare that the research was conducted in the absence of any commercial or financial relationships that could be construed as a potential conflict of interest.

## Publisher’s Note

All claims expressed in this article are solely those of the authors and do not necessarily represent those of their affiliated organizations, or those of the publisher, the editors and the reviewers. Any product that may be evaluated in this article, or claim that may be made by its manufacturer, is not guaranteed or endorsed by the publisher.

## Supplementary Material

Supplementary Figure 1 No tdTomato signal in the LNs from Rosa26-Tdtomato mice without LepR-Cre was found. Scale bar, 500μm.

Supplementary Figure 2
**(A)** iDISCO HEV imaging video in the WT LNs and db/db LNs. **(B)** Quantitative RT-PCR analysis was performed on whole LNs to examine expression of the tight junction markers ZO-1, claudin-5 and occludin in WT and db/db mice. The data are representative of two independent experiments with three mice/group. All the data are presented as mean ± SEM, Student’s t test, *p < 0.05, ***p < 0.001.

Supplementary Figure 3
**(A)** Dextran injections were used to assess HEV and lymphatic integrity. Scale bar, 50μm. Images are representative of five independent experiments (n=5). Quantification data with five mice/group (n=5) are summarized in bar chart. All the data are presented as mean ± SEM, Student’s t test, **p < 0.01. **(B)** ER-TR7 co-staining with HEVs and lymphatics to demonstrate differences in ER-TR7 deposition between leptin-treated ob/ob mice and control. Scale bar, 20μm (HEV), 50μm (LYVE1).

Supplementary Figure 4
**(A)** Flow cytometry profiling of LepR^+^ cell population changes in the LNs of control and CCL19^cre^;LepR^fl^ mice. Data from two independent experiments (n=5 mice/group) are summarized in the bar chart. ***p < 0.001, n.s.: not significant. **(B)** Fluorescence micrographs show differences in the microarchitecture between the LNs of WT;LepR^fl^ and CCL19^cre^;LepR^fl^ mice, as indicated by expression of ER-TR7, PDPN, collagen I and fibronectin. Scale bar, 50μm. Images are representative of two independent experiments from five mice/group (n=5). **(C)** Quantification data from two independent experiments with five mice/group (n=5) are summarized in bar chart. All the data are presented as mean ± SEM, Student’s t test, *p < 0.05, **p < 0.01. **(D)** Flow cytometric analysis of inflammatory parameters in WT;LepR^fl^ and CCL19^cre^;LepR^fl^ mouse LNs. Data from two independent experiments (n=5 mice/group/experiment) are summarized in bar chart. n.s., not significant. **(E)** Comparison of the size of DLNs in WT;LepR^fl^ and CCL19^cre^;LepR^fl^ mice LNs with CFA stimulation. **(F)** Flow cytometry profiling of PD-L1^+^ FRCs and CD8^+^IFNγ^+^ T cells in the LNs of WT;LepR^fl^ and CCL19^cre^;LepR^fl^ mice after CFA stimulation. Data from two independent experiments with five mice/group (n=5) are summarized in bar chart. All the data are presented as mean ± SEM, Student’s t test, n.s., not significant. **(G, H)** Flow cytometric analysis of T cell and B cell responses in CCL19^Cre^;LepR^fl^ and control mice immunized with CFA + OVA. Percentages of follicular T cells gated as CD4^+^CXCR5^+^ICOS^+^, Tfh cells as CD4^+^CXCR5^+^ICOS^+^FoxP3^-^, proliferating Tfh cells gated as CD4^+^CXCR5^+^ICOS^+^FoxP3^-^Ki67^+^ and plasma cells as CD138^hi^ (4-5 mice/group) are summarized in bar chart. n.s., not significant.
