# Supplementary figures and images for: Characterization of Leptin Receptor+ Stromal Cells in Lymph Node
Source: Front Immunol. 2022 Jan 17;12:730438. doi: 10.3389/fimmu.2021.730438 (PMC8801441; doi:10.3389/fimmu.2021.730438)

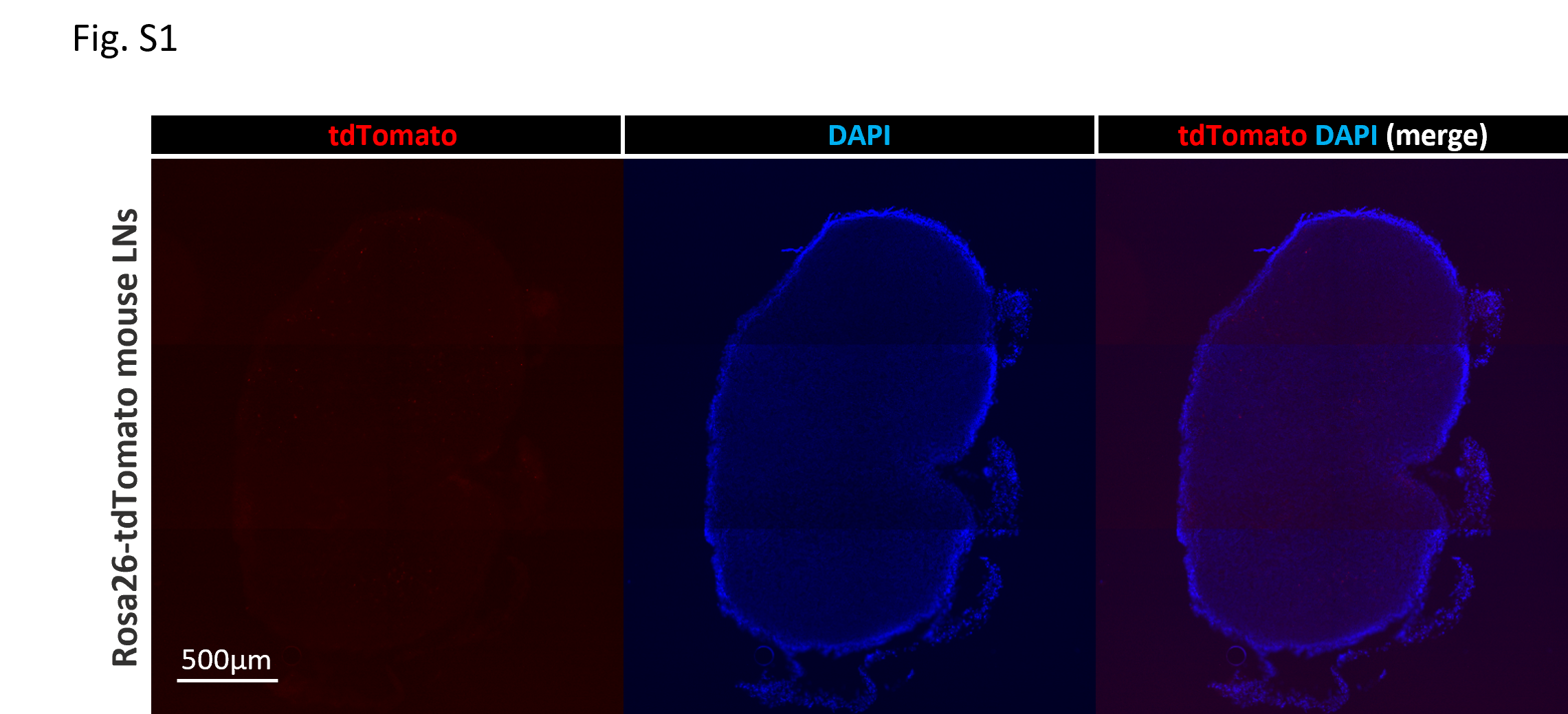

Supplement: Supplementary Figure 1 — No tdTomato signal in the LNs from Rosa26-Tdtomato mice without LepR-Cre was found. Scale bar, 500μm. [file Image_1.png]

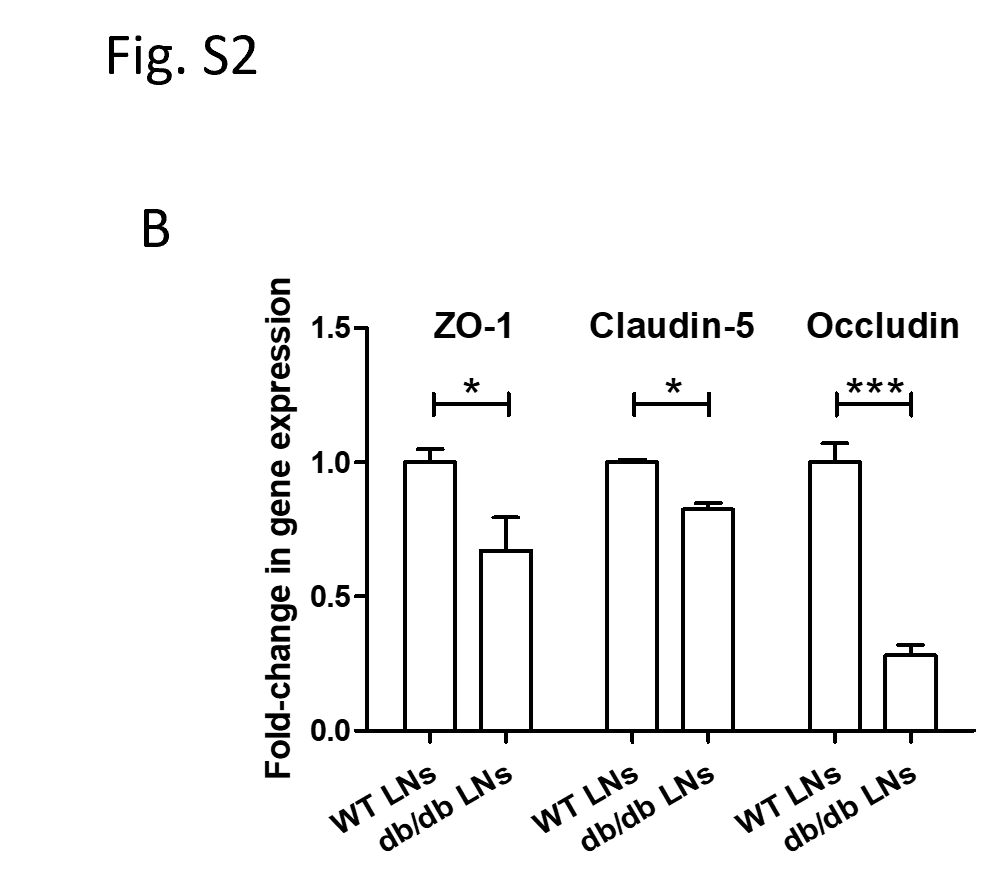

Supplement: Supplementary Figure 2 — (A) iDISCO HEV imaging video in the WT LNs and db/db LNs. (B) Quantitative RT-PCR analysis was performed on whole LNs to examine expression of the tight junction markers ZO-1, claudin-5 and occludin in WT and db/db mice. The data are representative of two independent experiments with three mice/group. All the data are presented as mean ± SEM, Student’s t test, *p < 0.05, ***p < 0.001. [file Image_2.png]

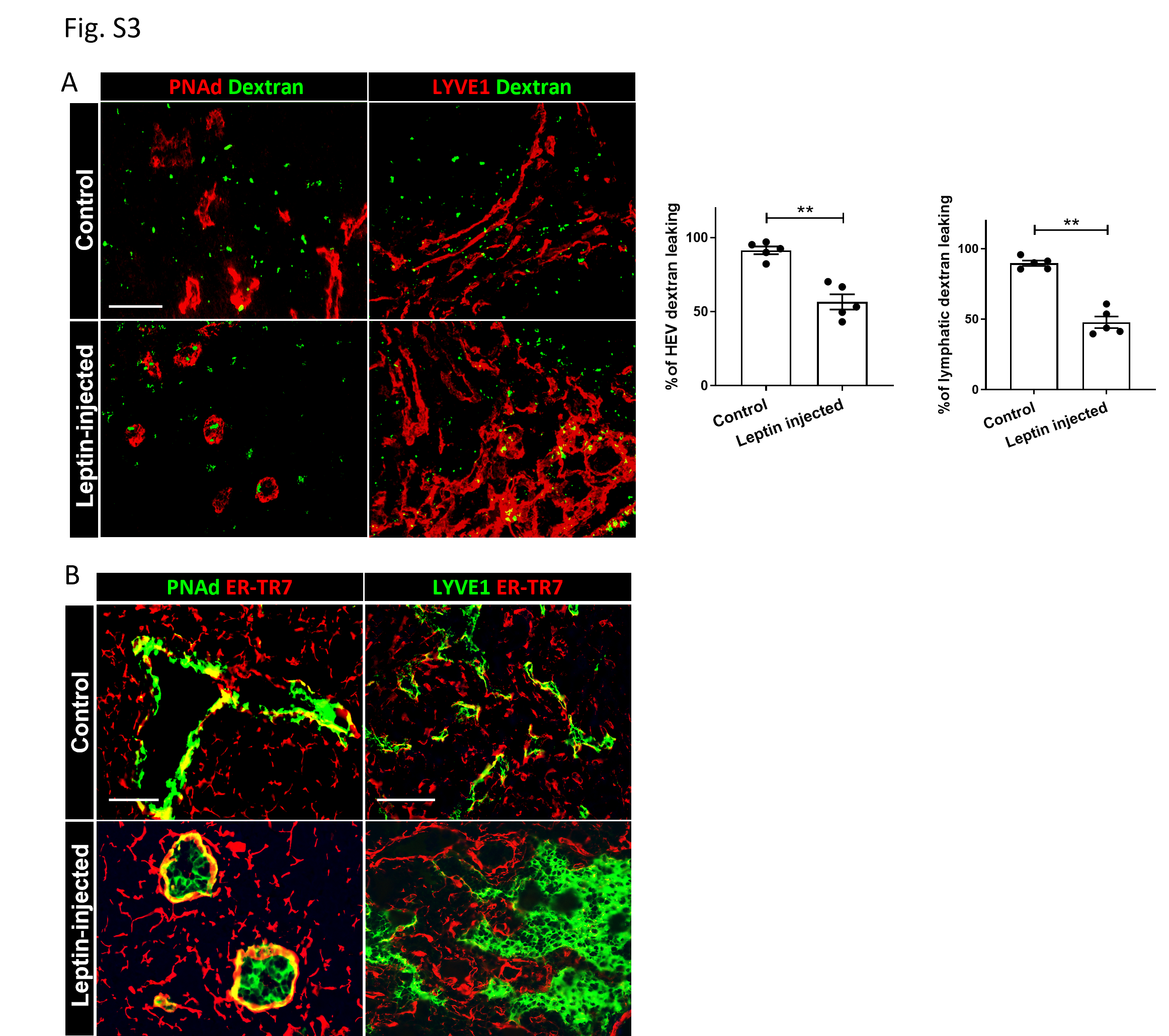

Supplement: Supplementary Figure 3 — (A) Dextran injections were used to assess HEV and lymphatic integrity. Scale bar, 50μm. Images are representative of five independent experiments (n=5). Quantification data with five mice/group (n=5) are summarized in bar chart. All the data are presented as mean ± SEM, Student’s t test, **p < 0.01. (B) ER-TR7 co-staining with HEVs and lymphatics to demonstrate differences in ER-TR7 deposition between leptin-treated ob/ob mice and control. Scale bar, 20μm (HEV), 50μm (LYVE1). [file Image_3.png]

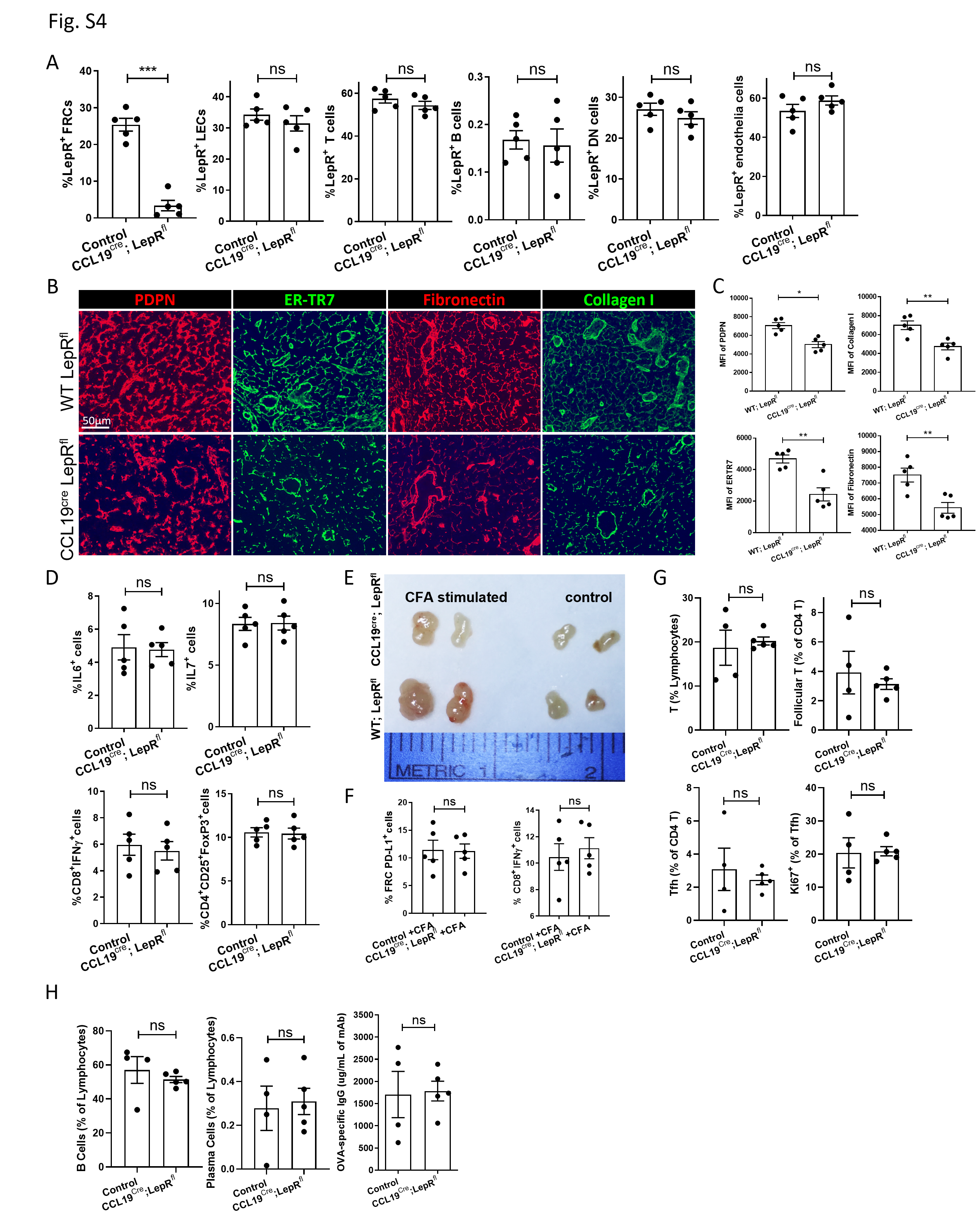

Supplement: Supplementary Figure 4 — (A) Flow cytometry profiling of LepR+ cell population changes in the LNs of control and CCL19cre;LepRfl mice. Data from two independent experiments (n=5 mice/group) are summarized in the bar chart. ***p < 0.001, n.s.: not significant. (B) Fluorescence micrographs show differences in the microarchitecture between the LNs of WT;LepRfl and CCL19cre;LepRfl mice, as indicated by expression of ER-TR7, PDPN, collagen I and fibronectin. Scale bar, 50μm. Images are representative of two independent experiments from five mice/group (n=5). (C) Quantification data from two independent experiments with five mice/group (n=5) are summarized in bar chart. All the data are presented as mean ± SEM, Student’s t test, *p < 0.05, **p < 0.01. (D) Flow cytometric analysis of inflammatory parameters in WT;LepRfl and CCL19cre;LepRfl mouse LNs. Data from two independent experiments (n=5 mice/group/experiment) are summarized in bar chart. n.s., not significant. (E) Comparison of the size of DLNs in WT;LepRfl and CCL19cre;LepRfl mice LNs with CFA stimulation. (F) Flow cytometry profiling of PD-L1+ FRCs and CD8+IFNγ+ T cells in the LNs of WT;LepRfl and CCL19cre;LepRfl mice after CFA stimulation. Data from two independent experiments with five mice/group (n=5) are summarized in bar chart. All the data are presented as mean ± SEM, Student’s t test, n.s., not significant. (G, H) Flow cytometric analysis of T cell and B cell responses in CCL19Cre;LepRfl and control mice immunized with CFA + OVA. Percentages of follicular T cells gated as CD4+CXCR5+ICOS+, Tfh cells as CD4+CXCR5+ICOS+FoxP3-, proliferating Tfh cells gated as CD4+CXCR5+ICOS+FoxP3-Ki67+ and plasma cells as CD138hi (4-5 mice/group) are summarized in bar chart. n.s., not significant. [file Image_4.png]

## Slide 1
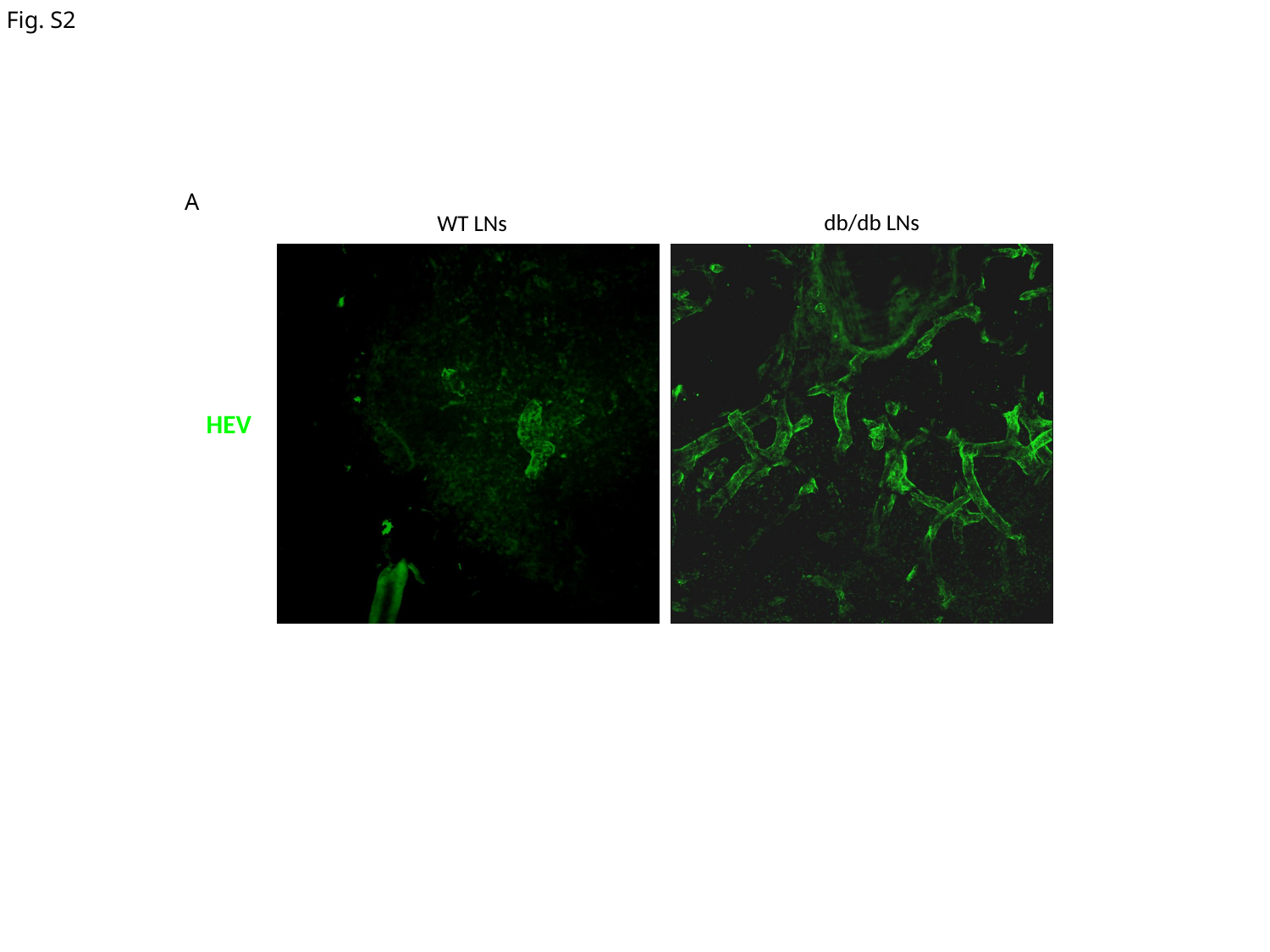

Fig. S2
A
db/db LNs
 WT LNs
HEV

Supplement: Supplementary file 5 [file Presentation_1.pptx]
